# Pyoderma gangrenosum, acne, and hidradenitis suppurativa (PASH) syndrome treated with upadacitinib

**DOI:** 10.1016/j.jdcr.2026.03.034

**Published:** 2026-03-20

**Authors:** Elika Javaheri, Stella X. Chen

**Affiliations:** a Mayo Clinic Alix School of Medicine, Phoenix, Arizona; b Department of Dermatology, Mayo Clinic, Scottsdale, Arizona

**Keywords:** autoinflammatory disorders, JAK inhibitors, PASH syndrome, upadacitinib

## Introduction

PASH syndrome is a rare autoinflammatory disorder characterized by the triad of pyoderma gangrenosum (PG), acne, and hidradenitis suppurativa (HS). Management is challenging as patients often exhibit variable responses to conventional therapies, such as corticosteroids, biologics, and antibiotics.^1^^,^^2^ This report describes a patient with PASH syndrome who achieved significant and sustained improvement following treatment with a Janus kinase (JAK) inhibitor.

## Case report

A 38-year-old woman with a history of polycystic ovarian syndrome, atopic dermatitis, nodulocystic acne since adolescence, and prediabetes presented with chronic, painful skin lesions. Physical examination revealed ulcerations, draining fistulas, and hypertrophic scarring involving the right axilla and inframammary folds. Discrete shallow ulcers with violaceous borders and cribriform scarring were noted on bilateral lower extremities. Acne affecting the face with atrophic scarring was observed.

Her dermatologic symptoms began 21 years earlier with recurrent draining lesions in the axillae. Biopsies of lower extremity ulcers demonstrated dense neutrophilic infiltrate with negative infectious workup, consistent with PG. Axillary lesions were clinically consistent with severe Hurley stage III HS. Genetic testing revealed heterozygous mutations in *NOD2* (c.2717+158C>T and c.2023C>T) and a variant in *SOCS1* (c.112_132del) supporting a diagnosis of PASH syndrome in the context of pyoderma gangrenosum, nodulocystic acne, and hidradenitis suppurativa. These alleles were not predicted to be pathologic but were considered risk alleles by the laboratory. The patient reported no family history of related symptoms; however, a significant portion of the family history was unavailable. To the knowledge of her care team, no family members received additional genetic testing based on these results. Screening for malignancy and inflammatory bowel disease (colonoscopy and negative gastrointestinal symptoms) was negative.

**Fig 1:**
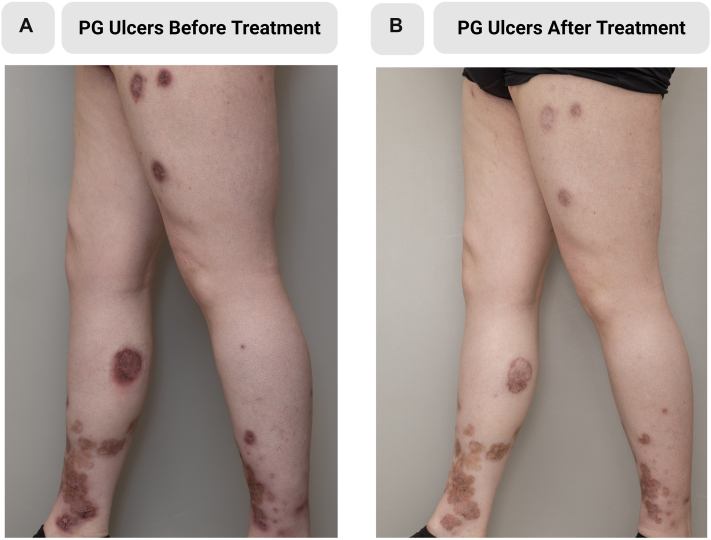
Pyoderma gangrenosum improvement following upadacitinib. At the initial visit, the patient presented with numerous painful ulcers with violaceous and undermined borders and cribriform scarring consistent with pyoderma gangrenosum **(A)**. 3 months of treatment with upadacitinib led to healed ulcers with scarring **(B)**.

**Fig 2:**
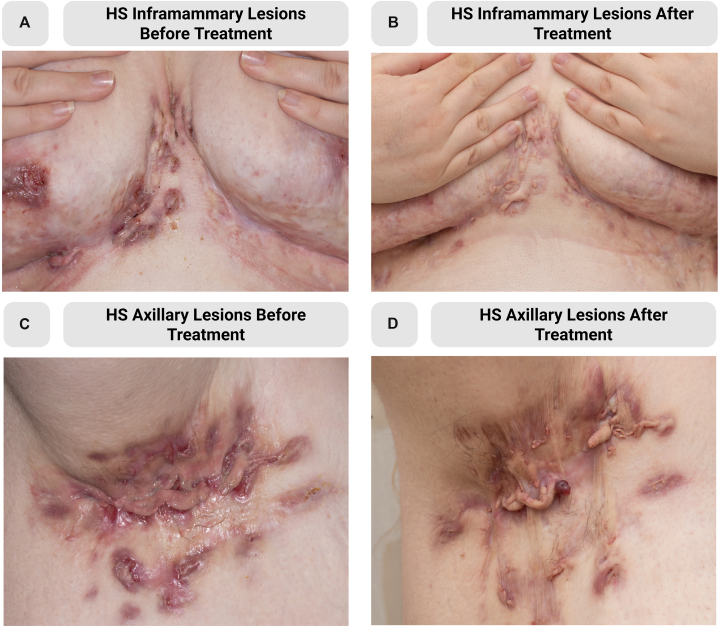
Hidradenitis Suppurativa Improvement Following Upadacitinib. Intermammary and inframammary folds **(A)** and axilla **(C)** with draining fistulas, hypertrophic scarring, inflammatory nodules, and erythematous borders. After 3 months of treatment, drainage and erythema improved while scarring remained **(B** and **D)**.

The patient had previously received multiple systemic therapies, including tetracyclines (unknown duration), adalimumab (8-month duration), etanercept (20 months), methotrexate (17 months), and mycophenolate (8 months). Adalimumab improved HS but not PG and was discontinued after anti-drug antibody formation. Infliximab was considered but was cost prohibitive. Given her concomitant atopic dermatitis and refractory PASH, upadacitinib 30 mg daily was initiated. After 3 months, she demonstrated marked improvement in PG and HS lesions (as demonstrated in Figs 1 and 2 respectively), with resolution of erythema, drainage, and ulcerations, and no adverse effects, including acne exacerbation. After 12 months of treatment, her PASH symptoms remained in remission and she underwent surgical excision of axillary HS.

## Discussion

PASH syndrome is a rare and heterogeneous autoinflammatory condition characterized by neutrophil-rich inflammation and overexpression of IL-1 family cytokines.^3^ Genetic studies have implicated variants in *PSTPIP1*, *NCSTN*, *PSENEN*, and *NOD2*, although these findings are inconsistent, suggesting a polygenic or multifactorial pathogenesis.^3^^,^^4^ In addition to *PASH*, *NOD2* mutations have been associated with other autoinflammatory conditions including inflammatory bowel disease, Yao syndrome, and Blau syndrome.

Interestingly, our patient was also found to have a heterozygous *SOCS1* variant of unknown significance. *SOCS1* negatively regulates the JAK-STAT (Signal Transducer and Activator of Transcription) pathway, and loss of *SOCS1* function results in overactivation of the JAK/STAT pathway, contributing to autoinflammatory states. While *SOCS1* mutations have not been reported in PASH syndrome or PG, they have been linked to inflammatory bowel disease.^5^ Previous *SOCS1* variants have been reported in association with a case of Crohn’s disease, psoriasis, pyoderma gangrenosum, and chronic rhinosinusitis (c58C>T) and a patient with asthma, severe unclassified polyarthritis, Sjögren’s syndrome, and chronic intestinal pseudo-occlusion (c.298_301dup).^5^ Thus, there is a wide spectrum of clinical phenotypes associated with *SOCS1* variants. Our patient was found to have a VUS, so we cannot determine whether, or to what extent, this variant contributed to her PASH presentation. The potential interaction between *SOCS1* and *NOD2* variants in this patient remains unclear but may have contributed to disease severity.

Management of PASH is difficult as it is frequently resistant to conventional therapies. Corticosteroids and immunosuppressants (cyclosporine, methotrexate) often provide only partial or temporary benefit.^6^ Biologics targeting tumor necrosis alpha (adalimumab, infliximab) and IL-1 (anakinra) have shown efficacy in some cases, but responses are variable and relapses are common. The JAK-STAT pathway mediates signaling for multiple pro-inflammatory cytokines, including IL-6, interferon gamma, and downstream mediators of IL-1 activity. Notably, JAK1 expression is elevated in PG and acne vulgaris lesions, whereas elevated JAK2 expression is not consistently associated with either.^7^^,^^8^ Therefore, it is possible JAK1 plays a central role in PASH syndrome – accordingly, the selectivity of upadacitinib for JAK1 is a favorable quality. Other JAK inhibitors such as tofacitinib have demonstrated efficacy in neutrophilic dermatoses and HS, making them attractive candidates for PASH.^9^ Their ability to target multiple cytokine pathways may explain the rapid and sustained improvement observed in our patient. Several clinical trials are currently evaluating JAK inhibitors for HS.

This case highlights the potential role of JAK inhibitors as a therapeutic option for refractory PASH syndrome, with sustained remission for over a year. These findings suggest broader therapeutic implications, as JAK inhibitors may also benefit related autoinflammatory PASH-adjacent syndromes such as PAPA (Pyogenic Arthritis, PG, and Acne) and PAPASH (Pyogenic Arthritis, PG, Acne, and HS).^10^

## Conflicts of interest

None disclosed.
